# Retinal changes in solid organ and bone marrow transplantation patients

**DOI:** 10.1590/S1679-45082017AO3992

**Published:** 2017

**Authors:** Fernando Korn Malerbi, Sergio Henrique Teixeira, Luis Gustavo Gondo Hirai, Nilson Hideo Matsudo, Adriano Biondi Monteiro Carneiro

**Affiliations:** 1 Hospital Israelita Albert Einstein, São Paulo, SP, Brazil.

**Keywords:** Transplantation, Bone marrow transplantation, Diabetic retinopathy, Immunosuppression, Central serous chorioretinopathy

## Abstract

**Objective:**

To evaluate retinal changes in patients who underwent solid organ or bone marrow transplantation.

**Methods:**

A retrospective analysis of medical records of patients evaluated from February 2009 to December 2016. All patients included underwent funduscopy. Clinical and demographic data regarding transplantation and ophthalmological changes were collected.

**Results:**

A total of 126 patients were analyzed; of these, 108 underwent transplantation and 18 were in the waiting list. Transplantation modalities were heart, lung, kidney, liver, pancreas, combined pancreas and kidney and bone marrow transplantation. The main pre-transplantation comorbidities were diabetes and arterial hypertension. Of the 108 transplanted patients, 82 (76%) had retinal changes. All patients who underwent pancreas or combined pancreas and kidney transplantation had diabetic retinopathy. The main retinal changes found were diabetic retinopathy, hypertensive retinopathy, retinal vascular occlusions, chorioretinal infections and central serous chorioretinopathy.

**Conclusion:**

Retinal changes were either related to preexisting conditions, mainly diabetic retinopathy, or developed postoperatively as a complication of the surgical procedure, or as an infection related to the immunosuppressive status, or due to drug toxicity. These patients may present with complex ophthalmological changes and should be carefully evaluated prior to surgery and further followed by an ophthalmologist skilled in the management of diabetic retinopathy and posterior pole infections.

## RESUMO

**Objetivo:**

Analisar as alterações retinianas de pacientes submetidos a transplantes de órgãos sólidos ou de medula óssea.

**Métodos:**

Análise de prontuário dos pacientes avaliados no período de fevereiro de 2009 a dezembro de 2016. Todos os pacientes incluídos foram submetidos à avaliação fundoscópica. Foram coletados dados demográficos e clínicos, referentes ao transplante e às alterações oftalmológicas encontradas.

**Resultados:**

Foram avaliados 126 pacientes, sendo 108 submetidos a transplantes e 18 que aguardavam o procedimento. Foram avaliados pacientes submetidos a transplantes de coração, pulmão, rim, fígado, pâncreas, pâncreas-rim e medula óssea. As principais comorbidades pré-transplante foram diabetes e hipertensão arterial. Dos 108 pacientes transplantados, 82 (76%) apresentaram alterações retinianas. Todos os pacientes submetidos ao transplante de pâncreas ou pâncreas-rim apresentaram alterações retinianas relacionadas ao diabetes. As principais alterações retinianas detectadas foram retinopatia diabética, retinopatia hipertensiva, oclusões vasculares retinianas, infecções coriorretinianas e coriorretinopatia serosa central.

**Conclusão:**

As alterações retinianas estavam relacionadas a doenças preexistentes, principalmente à retinopatia diabética, ou surgiram após o transplante, como complicação do procedimento cirúrgico, ou como complicação infecciosa associada à imunossupressão, ou ainda por toxicidade medicamentosa. Tais pacientes podem apresentar alterações oculares complexas, devendo ser submetidos à avaliação retiniana pré-operatória cuidadosa e ao acompanhamento pós-operatório por oftalmologista especializado no manejo da retinopatia diabética e de doenças infecciosas do segmento posterior ocular

## INTRODUCTION

Patients undergoing organ transplantation may present with ocular changes in the pre-, peri, or postoperative periods.^1^ These changes may be either related to the deterioration of retinal diseases occurring prior to transplantation, or represent complications of the procedure or the treatment started in the postoperative period. The surgical procedure for transplantation is usually extensive and may cause important hemodynamic changes that may reflect in the ocular perfusion. During the postoperative follow-up, these patients undergo immunosuppressive therapy, which may facilitate the onset of opportunistic infections whose target organs are ocular structures such as the retina and choroid.^2^ Additionally, the drugs used in the postoperative management may cause retinal damage due to toxicity or lead to the development of retinal diseases due to secondary metabolic changes.^1^

The *Hospital Israelita Albert Einstein* (HIAE) has a large solid organ and bone marrow transplantation program. The Diagnostic Center in Ophthalmology (CDOF - *Centro de Diagnóstico em Oftalmologia*) team of the *Instituto Israelita de Responsabilidade Social* performs eye evaluations of several patients in this program.

## OBJECTIVE

To analyze retinal changes in patients undergoing solid organ or bone marrow transplantation.

## METHODS

This is a retrospective study based on the analysis of medical records. Clinical and demographic data from patients in the transplantation program examined by the CDOF team, from February 2009 to December 2016, were collected. Both patients groups - those undergoing transplantation (Transplantation Group) and those awaiting for the procedure (Waiting List Group) were evaluated. All patients included underwent funduscopic exam performed by one single retina specialist ophthalmologist.

Data regarding sex; age at transplantation; transplanted organ; indication for transplantation; preoperative clinical characteristics; postoperative clinical course; time elapsed between transplantation and ophthalmological assessment; findings in retinal exam; and results of other ophthalmological tests performed were analyzed. Data regarding the ophthalmological treatment given to patients with changes and the outcome of these treatments were also considered.

Funduscopic examination was performed on an outpatient basis whenever possible; however, it was sometimes performed in-hospital, at bedside. This consisted of binocular indirect ophthalmoscopy plus fundus biomicroscopy in a slit lamp whenever possible. In selected cases, additional assessment by means of ancillary tests was performed.

This study was approved by the Research Ethics Committee, of the *Instituto Israelita de Ensino e Pesquisa do Hospital Israelita Albert Einstein*, under number 1.678.157 and CAAE: 57986416.3.0000.0071. Since this was a retrospective study based on the analysis of medical records with no contact with participants, no Informed Consent Form (ICF) was deemed necessary by the committee.

The descriptive statistical analysis was carried out using Microsoft Excel for Mac 14.0.0 (Microsoft Corporation, California, USA).

## RESULTS

A total of 126 patients were analyzed, in that, 108 (86%) were from Transplantation Group and 18 (14%) from Waiting List Group; retinal changes were found in 97 patients.

In Transplantation Group, 61 (56%) patients were males. The mean age at the examination was 50±13 years, and the average interval from transplantation to examination was 35±37 months. There was a long interval between transplantation and ocular assessment in some patients, with a maximal interval of 142 months, which caused a high standard deviation. However, no negative values were found. In Waiting List Group, 12 (67%) patients were males. The mean age at the examination was 50±14 years.

The types of transplantation performed and the conditions leading to transplanted organ failure are described in table 1.

**Table 1:** Types of transplantations performed and causes

Transplanted organ	Cause of organ failure	n (%)
Heart (n=3)	Idiopathic	1 (33)
	Dilated cardiomyopathy	2 (66)
Liver (n=41)	Primary sclerosing cholangitis	2 (5)
	Alcoholic cirrhosis	4 (9)
	Familial amyloidosis	2 (5)
	Hepatitis B	1 (2)
	Hemochromatosis	1 (2)
	Hepatitis C	21 (51)
	Autoimmune hepatitis	4 (9)
	Cryptogenic cirrhosis	5 (12)
	Fulminant hepatitis	2 (5)
Pancreas (n=1)	*Diabetes mellitus*	1 (100)
Combined pancreas and kidney (n=21)	*Diabetes mellitus*	21 (100)
Lung (n=3)	Cystic fibrosis	1 (33)
	Bronchiectases	1 (33)
	Chronic obstructive pulmonary disease	1 (33)
Bone marrow (n=3)	Leukemia	2 (66)
	Adrenoleukodystrophy	1 (33)
Kidney (n=36)	Polycystic kidney disease	1 (3)
	*Diabetes mellitus*	15 (42)
	Primary hyperoxaluria	1 (3)
	Indeterminate	5 (14)
	Membranoproliferative glomerulonephritis	2 (5)
	Neurogenic bladder	1 (3)
	Systemic arterial hypertension	9 (25)
	Focal segmental glomerulosclerosis	1 (3)
	Chronic glomerulonephritis	1 (3)

The major pre-transplantation comorbidities, *i.e*., conditions existing prior to transplantation and not related to transplanted organ failure, were arterial hypertension, which was present in 20 (18%) patients, and *diabetes mellitus* in 12 (11%).

The major non-ocular post-transplantation associated diseases were classified as infectious and noninfectious comorbidities. In the first group, the following infections were diagnosed, according to the clinical, laboratory and radiological context: cytomegalovirus (laboratory diagnosis using antigenemia and polymerase chain reaction – PCR) in 27 (25%) patients; sepsis in 5 (5%); tuberculosis in 4 (4%) patients (clinical radiological diagnosis); herpes zoster in 2 (2%) patients (clinical laboratory diagnosis with serologic tests); neurotoxoplasmosis in 2 (2%) patients; and herpetic encephalitis in 1 (1%) patient. In relation to noninfectious comorbidities, 23 (21%) patients developed post-transplantation systemic arterial hypertension; 20 (19%), *diabetes mellitus*; 9 (8%), heart diseases (coronary insufficiency, acute myocardial infarction or congestive heart failure); 8 (7%), stroke; 2 (2%) chronic obstructive pulmonary disease; and 2 (2%), lymphoma.

The major acute or chronic complications of transplanted patients during the follow-up period were surgical complications in the immediate postoperative period (2; 2%); graft loss (25; 23%); kidney failure in patients not originally receiving kidney transplantation (12; 11%); liver failure in patients not originally receiving liver transplantation (1; 1%); and death (12; 11%).

The main reasons leading to ocular evaluation were complaints of poor visual acuity and clinical investigation for staging systemic diseases, or as part of the investigation of infectious diseases.

Patients were evaluated on an outpatient basis whenever possible: 94 patients from Transplantation Group (87%) and 15 patients from Waiting List Group (83%); however, some patients were evaluated at bedside (14 in Transplantation Group and 3 in Waiting List Group).

A considerable number of patients underwent more than one ophthalmological evaluation: 49 (45%) patients of Transplantation Group and 7 (39%) of Waiting List Group. In addition to the clinical ophthalmological evaluation, some cases also required ancillary tests, mainly ocular ultrasound (7; 6%), fluorescein angiography (7; 6%) and optical coherence tomography (6; 6%).

Several patients underwent ophthalmological procedures during the follow-up period. The major procedures were cataract extraction (16; 15%), panretinal laser photocoagulation (10; 9%) and pars plana vitrectomy (6; 6%).

Of the 108 patients undergoing transplantation, 82 (76%) showed several retinal changes and 26 had a normal retinal evaluation. The latter were distributed as follows: heart transplantation (1 patient); liver transplantation (13 patients), kidney transplantation (6 patients); lung transplantation (3 patients); and bone marrow transplantation (3 patients). All patients undergoing pancreas or combined pancreas-kidney transplantation showed *diabetes mellitus*-related retinal changes, some of them presenting also other retinal conditions.

In patients evaluated prior to transplantation (Waiting List Group), diabetic retinopathy was the most frequent change (9; 50%), and eight of them had the proliferative form. The second most frequent retinal change in Waiting List Group was hypertensive retinopathy, which was present in four patients (22%).

Table 2 describes retinal changes according to the type of transplantation performed, whereas table 3 shows the classification according to the type of retinal change.

**Table 2:** Retinal changes by transplanted organ*

Transplant	Changes	n (%)
Heart	Proliferative diabetic retinopathy	1 (33)
	Central serous chorioretinopathy	1 (33)
Liver	Hypertensive retinopathy	12 (29)
	Non-proliferative diabetic retinopathy	4 (10)
	Proliferative diabetic retinopathy	4 (10)
	Rhegmatogenous retinal detachment^†^	2 (5)
	Endogenous endophthalmitis	2 (5)
	Non-diabetic vitreous hemorrhage	1 (2)
	Retinal vascular occlusion	2 (5)
	Toxoplasmic chorioretinitis	2 (5)
	Cytomegalovirus retinitis	2 (5)
	Panuveitis	1 (2)
	Age-related macular degeneration^†^	1 (2)
	Retinitis pigmentosa^†^	1 (2)
	Central serous chorioretinopathy	1 (2)
Pancreas	Proliferative diabetic retinopathy	1 (100)
	Hypertensive retinopathy	1 (100)
Pancreas-kidney	Proliferative diabetic retinopathy	20 (95)
	Non-proliferative diabetic retinopathy	1 (5)
	Hypertensive retinopathy	2 (9)
	Retinal vascular occlusion	3 (14)
	Central serous chorioretinopathy	1 (5)
	Toxoplasmic chrorioretinitis	1 (5)
Kidney	Hypertensive retinopathy	15 (42)
	Proliferative diabetic retinopathy	10 (28)
	Non-proliferative diabetic retinopathy	2 (5)
	Retinal vascular occlusion	2 (5)
	Panuveitis	1 (3)
	Central serous chorioretinopathy	1 (3)

* More than one change could be present in the same patient, thus resulting in sums higher than 100%; ^†^ Retinal changes existing prior to transplantation and with clinical course independent of transplantation.

**Table 3:** Retinal changes in patients undergoing transplantation*

Type of change	n	Time between transplantation and ocular examination	

		Mean (months)	Range (months)
Infectious, (%)	7 (6)	-	-
Chorioretinitis (toxoplasmosis)	3	16	1-111
Endogenous endophthalmitis	2	26	1-51
Cytomegalovirus retinitis	2	1	0-1
Diabetic retinopathy, (%)	45 (42)	-	-
Proliferative diabetic retinopathy	38	42	1-142
Non-proliferative diabetic retinopathy	7	55	1-130
Hypertensive retinopathy^†^, (%)	30 (28)	32	1-115
Vascular occlusions, (%)	7 (6)	61	5-130
Noninfectious panuveitis^‡^, (%)	2 (2)	13	12-13
Central serous chorioretinopathy, (%)	4 (4)	19	1-60
Miscellaneous, (%)	12 (11)	-	-
Rhegmatogenous retinal detachment	5	41	3-111
Age-related macular degeneration	1	5	-
Non-diabetic vitreous hemorrhage	1	81	-
Retinitis pigmentosa	1	13	-
Epiretinal membrane	4	54	10-95

* More than one change could be present in the same patient; ^†^ one patient had Purtscher-like retinopathy secondary to a hypertensive crisis; ^‡^ one patient had choroidal detachment secondary to cidofovir.

**Figure 1:**
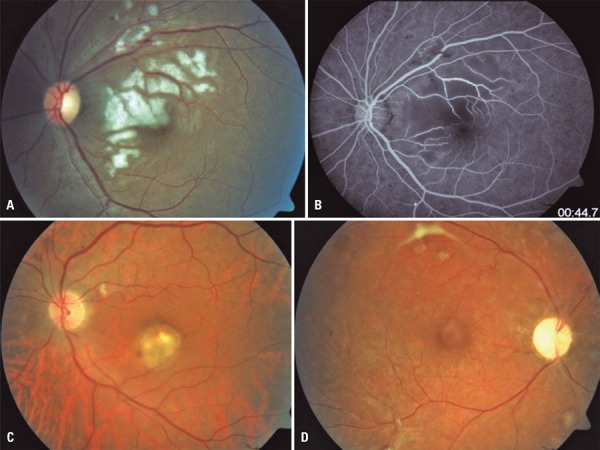
Retinal complications in transplantation patients

Some of the retinal changes present after transplantation may be considered complications of transplantation, or related to the immunosuppressive therapy. These complications are described in table 4, and some selected cases are shown in figure 1.

**Table 4:** Retinal changes in patients undergoing transplantation*

Type of complication	Transplanted organ	n
Diabetic retinopathy^†^		
Vitreous hemorrhage	Pancreas-kidney	10
	Kidney	2
Tractional retinal detachment	Pancreas-kidney	2
	Kidney	2
Neovascular glaucoma	Liver	1
Diabetic papillopathy	Liver	1
Infectious		
Toxoplasmic chorioretinitis	Liver	2
	Pancreas-kidney	1
Endophthalmitis	Liver	2
Cytomegalovirus retinitis	Liver	2
Retinal vascular occlusion^‡^	Kidney	2
	Pancreas-kidney	3
	Liver	2
Other complications		
Panuveitis^§^	Kidney	1
Purtscher-like^¶^	Kidney	1
Central serous chorioretinopathy	Heart	1
	Kidney	1
	Liver	1

* More than one complication could be present in the same patient; ^†^ deterioration of preexisting diabetic retinopathy;

^‡^ procedure-related; ^§^ cidofovir-related toxicity; ^¶^ procedure-related, complication of Hypertensive Retinopathy.

(A and B) Purtscher-like retinopathy secondary to hemodynamic instability (hypertensive crisis) on postoperative day 8 of renal transplantation (A, color retinography; B, red-free retinography). (C) Color retinography shows macular lesion secondary to toxoplasmic chorioretinitis in a liver-transplanted patient undergoing immunosuppression. This case presentation was atypical, with mild vitreous reaction. (D) Central retinal artery occlusion after hemodynamic instability (hypovolemic shock) in patient receiving combined pancreas-kidney transplantation.

**Figure 2:**
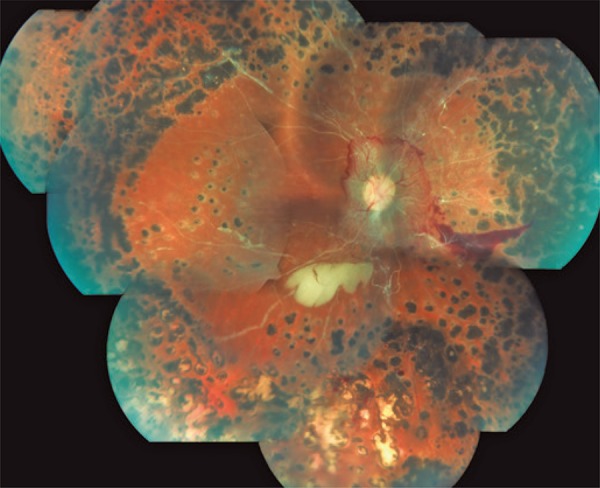
Diabetic retinopatihy in a combined pancreas-kidney transplantation recipient

**Figure 3:**
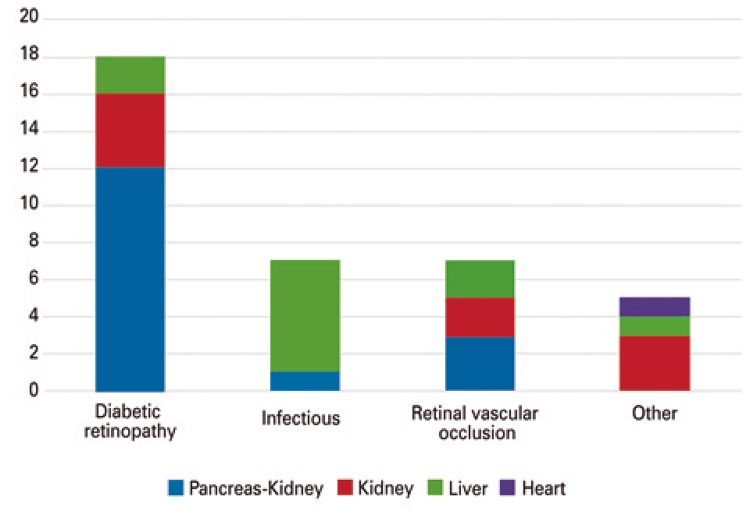
Retinal complications grouped by type of complication and transplantation

Other retinal changes may be considered as deterioration of preexisting conditions. Figure 2 depicts a case of proliferative diabetic retinopathy treated with panretinal laser photocoagulation with extensive ischemia and signs of proliferative activity in a patient receiving combined pancreas-kidney transplantation. Figure 3 shows retinal complications grouped by type of complication and type of transplantation.

Panoramic retinography of a 29-year-old patient undergoing combined pancreas-kidney transplantation showing end-stage diabetic retinopathy, with presence of neovascular activity despite extensive panretinal photocoagulation. Vascular sheathing indicative of retinal ischemia can also be observed. The other eye of the patient showed amaurosis resulting from diabetic retinopathy.

## DISCUSSION

The present study showed that the major retinal complications in transplanted patients were deterioration of diabetic retinopathy (or new diabetic retinopathy due to onset of post-transplantation diabetes); opportunistic infection-related complications due to immunosuppression; and retinal vascular occlusions associated to hypertensive retinopathy or intra- or perioperative hemodynamic fluctuations.

Some of the retinal changes observed may be considered transplant-related complications. Infectious complications related to the immunosuppressive therapy are considered the major and most severe retinal complications in patients undergoing transplantation, and cytomegalovirus is considered the opportunistic agent more commonly causing retinal infection in transplant recipients.^1-3^ Transplanted patients using immunosuppresive drugs may present with atypical infectious retinal changes, as is the case of the patient with toxoplasmic posterior uveitis with no vitreous reaction reported here. Among the noninfectious complications, we should mention retinal vascular occlusions, central serous chorioretinopathy, retinal vasculitis, and drug toxicity.^1,2^

Diabetes and hypertension were the main conditions present in the preoperative period, either as the cause of organ failure or otherwise: all patients undergoing pancreas or combined pancreas-kidney transplantation in the present study had diabetic retinopathy in the preoperative period – many of whom with the most severe form, proliferative retinopathy. Thus, retinal complications associated with these conditions may be expected in this group of patients. Previously existing diabetic retinopathy may worsen, remain stable or improve,^4-8^ and post-transplantation blood glucose control does not halt its progression, especially in patients not receiving adequate specific treatment in the preoperative period.^4^

There is a consensus on the importance of the preoperative evaluation and treatment, as well as of a careful postoperative follow-up,^4^ especially in first 12 months following transplantation.^7^ Proper treatment is believed to ensure good visual function in the majority of patients.^5^

The retinal vascular occlusions observed in patients undergoing transplantation may be largely attributed to hypertensive retinopathy, either preexisting in the preoperative period or starting after transplantation.^9^ The presence of risk factors (diabetes, arterial hypertension and dyslipidemia) for retinal vascular occlusions should also be emphasized in patients receiving transplantation.^1,2,9^

Other conditions associated with retinal vascular occlusions include hypercoagulable states, venous stasis, increased inflammatory factors, blood transfusion, and toxicity of immunosuppressive drugs.^9-12^

In addition to infectious complications and retinal vascular occlusions, central serous chorioretinopathy is one of the retinal changes frequently reported in patients undergoing transplantation. Factors associated with this condition include the use of corticosteroid, hemodynamic fluctuations, stress, anxiety, and toxicity of immunosuppressive drugs, such as cyclosporine and tacrolimus.^13,14^

Drug toxicity may also affect patients undergoing transplantation, as in the case of the patient who presented with panuveitis with choroidal detachment and hypotony, associated with the use of the antiviral drug cidofovir for the treatment of cytomegalovirus infection. In the analysis of a similar case, the picture was attributed to ciliary body necrosis.^15^ Another uncommon retinal change was Purtscher-like retinopathy, whose pathophysiology may be associated with localized forms of retinal vasculitis and/or coagulation disorders,^16^ secondary to vascular alterations resulting from the surgical procedure, such as micro-occlusions of the retinal pre-capillary arterioles.^17^ Purtscher-like retinopathy was infrequently described after transplantation in patients with chronic kidney failure, as the initial presentation of retinal microvasculopathy after bone marrow transplantation, or associated with renal graft rejection.^18,19^ The present case is believed to have occurred secondarily to a hypertensive crisis on postoperative day 8 of kidney transplantation, characterizing the first case described in the literature with such an association.

In addition to the conditions herein described, other complications associated with poor visual acuity, which have not been observed in this case series, may occur after transplantation, including retinal microvasculopathy after bone marrow transplantation related to the use of immunosuppressive drugs;^20-23^ tacrolimus-associated ischemic optic neuropathy;^24,25^ tacrolimus-associated maculopathy;^26^ optical nerve toxicity for cyclosporine;^27^ and reversible posterior leukoencephalopathy syndrome related to the use of the immunosuppressants cyclosporine and tacrolimus^28,29^ or to the use of immnosuppressants associated with vascular injury.^30^

From the ophthalmological view point, many of the patients evaluated in this series presented with complex changes, and underwent more than one ophthalmological evaluation and ancillary tests. Several ophthalmological procedures were performed, mainly cataract extraction. In addition to the use of corticosteroids in many patients prior to transplantation, due to their underlying condition, most of the transplanted individuals undergo corticoid therapy in the postoperative period, and this contributes for cataract to be the most common ocular complication in transplanted patients;^10^ also, deterioration of cataract was reported in diabetic patients after transplantation.^4^ From a systemic view point, these patients are potentially severely ill, and many progressed to death, mainly due to infectious complications, graft rejection and/or failure. Thus, in addition to counting on multidisciplinary clinical and surgical teams directly related to the surgical procedure and postoperative management, a transplantation service should be prepared to treat highly complex ophthalmological cases.

Among the study limitations, we should mention its retrospective character and the analysis of information from a single center. This was a convenience sample, and many retinal changes existing prior to transplantation could not be quantified, which made it impossible to determine the progression of such changes after transplantation. However, given the number of patients assessed, the diversity of transplants performed, and the fact that the information had been collected from a referral center for transplantation, we believe that we were able to obtain a good characterization of retinal changes in this group of patients, and that these findings can be extrapolated to other transplantation centers.

## CONCLUSION

The post-transplantation retinal changes found may be related to preexisting conditions, mainly deterioration of diabetic retinopathy, or to complications of the surgical procedure, opportunistic infections, or drug toxicity. Patients in the transplantation program should undergo careful pre- and postoperative retinal assessment whenever possible, preferably by an ophthalmologist skilled in the management of diabetic retinopathy and infectious diseases of the posterior pole.

## References

[1] Chung H, Kim KH, Kim JG, Lee SY, Yoon HY (2007). Retinal complications in patients with solid organ or bone marrow transplantations. Transplantation.

[2] Ng P, McCluskey P, McCaughan G, Glanville A, MacDonald P, Keogh A (1998). Ocular complications of heart, lung, and liver transplantation. Br J Ophthalmol.

[3] Hashemi SB, Shishegar M, Nikeghbalian S, Salahi H, Bahador A, Kazemi K (2009). Endogenous Aspergillus endophthalmitis occurring after liver transplantation: a case report. Transplant Proc.

[4] Chow VC, Pai RP, Chapman JR, O’Connell PJ, Allen RD, Mitchell P (1999). Diabetic retinopathy after combined kidney-pancreas transplantation. Clin Transplant.

[5] Friberg TR, Tzakis AG, Carroll PB, Starzl TE (1990). Visual improvement after long-term success of pancreatic transplantation. Am J Ophthalmol.

[6] Giannarelli R, Coppelli A, Sartini M, Aragona M, Boggi U, Vistoli F (2005). Effects of pancreas-kidney transplantation on diabetic retinopathy. Transpl Int.

[7] Pearce IA, Ilango B, Sells RA, Wong D (2000). Stabilisation of diabetic retinopathy following simultaneous pancreas and kidney transplant. Br J Ophthalmol.

[8] Shipman KE, Patel CK (2009). The effect of combined renal and pancreatic transplantation on diabetic retinopathy. Clin Ophthalmol.

[9] Chung H, Yoon SY, Kim JG, Yoon YH (2009). Central retinal vein occlusion after liver transplantation. Acta Ophthalmol.

[10] Ates O, Keles M, Uyanik A, Bilen H, Cetinkaya R, Turkeli M (2008). Central retinal vein thrombosis and hyperhomocysteinemia in a young patient with renal transplantation. Transplant Proc.

[11] Izbicki G, Bairey O, Shitrit D, Lahav J, Kramer MR (2006). Increased thromboembolic events after lung transplantation. Chest.

[12] Donnadieu B, Amouyal F, Hoffart L, Matonti F (2014). Central retinal vein occlusion-associated tacrolimus after liver transplantation. Transplantation.

[13] Oliaei F, Rasoulinejad A, Seifi B (2007). An ophthalmological complication: central serous chorioretinopathy in a renal transplant recipient. Transplant Proc.

[14] Singh AD, Demirci H, Shields CL, Shields JA (2003). Central serous chorioretinopathy as a complication of postcardiac transplant corticosteroid therapy. Eye (Lond).

[15] Rapp P, Pilon F, Chiambaretta F, Ménérath JM, Jacomet C (2003). Bilateral uveitis with definitive hypotony caused by systemic cidofovir. J Fr Ophthalmol.

[16] Agrawal A, McKibbin MA (2006). Purtscher’s and Purtscher-like retinopathies: a review. Surv Ophthalmol.

[17] Miguel AI, Henriques F, Azevedo LF, Loureiro AJ, Maberley DA (2013). Systematic review of Purtscher’s and Purtscher-like retinopathies. Eye (Lond).

[18] Parc C (2007). Purtscher-like retinopathy as an initial presentation of a thrombotic microangiopathy associated with antineoplastic therapy. Am J Hematol.

[19] Lai WW, Chen AC, Sharma MC, Lam DS, Pulido JS (2005). Purtscher-like retinopathy associated with acute renal allograft rejection. Retina.

[20] Bylsma GW, Hall AJ, Szer J, West R (2001). Atypical retinal microvasculopathy after bone marrow transplantation. Clin Exp Ophthalmol.

[21] Cunningham ET, Irvine AR, Rugo HS (1996). Bone marrow transplantation retinopathy in the absence of radiation therapy. Am J Ophthalmol.

[22] Gloor B, Gratwohl A, Hahn H, Kretzschmar S, Robert Y, Speck B (1985). Multiple cotton wool spots following bone marrow transplantation for treatment of acute lymphatic leukaemia. Br J Ophthalmol.

[23] Webster AR, Anderson JR, Richards EM, Moore AT (1995). Ischaemic retinopathy occurring in patients receiving bone marrow allografts and campath-1G: a clinicopathological study. Br J Ophthalmol.

[24] Gupta M, Bansal R, Beke N, Gupta A (2012). Tacrolimus-induced unilateral ischaemic optic neuropathy in a non-transplant patient. BMJ Case Rep.

[25] Lake DB, Poole TR (2003). Tacrolimus. Br J Ophthalmol.

[26] Koh T, Baek SH, Han JI, Kim US (2011). Maculopathy associated with tacrolimus (FK 506). Korean J Ophthalmol.

[27] López-Jiménez J, Sánchez A, Fernández CS, Gutiérrez C, Herrera P, Odriozola J (1997). Cyclosporine-induced retinal toxic blindness. Bone Marrow Transplant.

[28] Gera DN, Patil SB, Iyer A, Kute VB, Gandhi S, Kumar D (2014). Posterior reversible encephalopathy syndrome in children with kidney disease. Indian J Nephrol.

[29] Tamhankar MA, Lesniak SP, Nallasamy S, Woo JH (2016). Tacrolimus-induced cerebral blindness in a liver transplant patient. Indian J Ophthalmol.

[30] Rao NM, Raychev R, Kim D, Liebeskind DS (2012). Elucidating the mechanism of posterior reversible encephalopathy syndrome: a case of transient blindness after central venous catheterization. Neurologist.

